# Clinical Practice and Safety of Endoscopic Balloon Dilation for Crohn's Disease–Related Strictures: A Nationwide Claim Database Analysis in Japan

**DOI:** 10.1155/2024/1291965

**Published:** 2024-11-14

**Authors:** Rintaro Moroi, Kunio Tarasawa, Hiroshi Nagai, Yusuke Shimoyama, Takeo Naito, Hisashi Shiga, Shin Hamada, Yoichi Kakuta, Kiyohide Fushimi, Kenji Fujimori, Yoshitaka Kinouchi, Atsushi Masamune

**Affiliations:** ^1^Division of Gastroenterology, Tohoku University Hospital, Sendai, Japan; ^2^Department of Health Administration and Policy, Tohoku University Graduate School of Medicine, Sendai, Japan; ^3^Department of Health Policy and Informatics, Tokyo Medical and Dental University Graduate School of Medicine and Dental Sciences, Bunkyo, Japan

**Keywords:** anastomotic stricture, big data, Crohn's disease, endoscopic balloon dilation, ulcerative colitis

## Abstract

**Introduction:** Although endoscopic balloon dilation (EBD) is a common therapeutic approach for managing strictures associated with Crohn's disease (CD), the clinical application and complication rates of EBD remain unclear.

**Methods:** We collected admission data for patients who underwent EBD using a nationwide database. We compared EBD outcomes between ileal and colonic strictures, CD and ulcerative colitis, and CD and anastomotic strictures arising from cancer-related surgery. Subsequently, propensity score matching was employed to facilitate comparisons between each group.

**Results:** The median duration of hospital stay was 4 days. Endoscopic hemostasis and urgent surgery rates after EBD for CD-related strictures were considerably low (0.035% and 0.11%, respectively). Most patients with CD underwent only one EBD procedure during a single admission. Although no significant differences in patient backgrounds and severe complications were observed between ileal and colonic stricture in CD, multiple EBD procedures were more commonly performed for ileal strictures than for colonic stricture. Moreover, EBD for ileal stricture was more frequently conducted in high-volume centers than in low-volume centers. Regarding severe complications after EBD, no significant differences were observed between CD-related strictures and ulcerative colitis or anastomotic strictures related to cancer surgery.

**Conclusion:** Our findings support the safe and effective use of EBD for both ileal and colonic strictures associated with CD. The clinical practice and safety outcomes of EBD for CD-related strictures were comparable to those for strictures stemming from other etiologies.

## 1. Introduction

Intestinal stricture is a common cause of bowel obstruction, with its occurrence linked to several diseases and procedures, including Crohn's disease (CD) [1], ulcerative colitis (UC) [2], and post–colorectal tumor surgery anastomosis [3]. Endoscopic balloon dilation (EBD) is the most widely employed procedure for alleviating stenotic conditions [4–6]. EBD reportedly demonstrates high technical and clinical success rates and low complication rates [4, 5, 7]. Moreover, CD guidelines have underscored the significance of EBD in addressing intestinal strictures associated with CD [6].

However, the clinical application of EBD, involving factors such as complication rates, hospital stay duration, and the frequency of EBD procedures during a single hospital admission, remains unclear. Furthermore, distinctions between CD and other diseases and situations (e.g., CD vs. anastomotic stricture after intestinal cancer surgery or ileal vs. colonic strictures in patients with CD) remain unexplored. The limited number of patients who undergo EBD at a single institution makes it challenging to analyze complication rates, which are expected to be low. A systematic review reported a complication (bleeding and perforation) rate of only 1.82% for EBD performed to treat small intestinal strictures in patients with CD [7].

We have previously reported clinical research that employed a nationwide claim database [8, 9], known as Diagnosis Procedure Combination (DPC). It contains many patients' data, enabling the analysis of rare diseases and complications.

In this study, we aimed to elucidate the clinical practice and severe complication rates of EBD for CD using a nationwide database in Japan. We intended to compare UC and anastomotic strictures caused by small bowel and colorectal cancers.

## 2. Methods

### 2.1. DPC Data System

The DPC database is a medical claims database for inpatient and acute-care hospitals in Japan. In 2018, the database encompassed approximately 83% of acute-care beds within 1730 hospitals [10]. There are six distinct categories of diagnosis in the DPC database: main diagnosis, main disease triggering admission, most resource-consuming diagnosis, second most resource-consuming diagnosis, comorbidities at admission, and complications after admission. The database also includes patient demographics, including sex, age, body mass index (BMI), smoking history (Brinkman index), and Charlson comorbidity index (CCI). Additionally, it includes information regarding medical procedures (surgery and endoscopic hemostasis), hospital volume (the average number of EBD procedures conducted annually at each institution), medical costs, and duration of hospital stay. The accuracy of disease classification using International Classification of Diseases Tenth Revision (ICD-10) codes for CD and UC had been validated [11].

### 2.2. Extraction of Eligible Admissions and Data Collection

**Figure 1:**
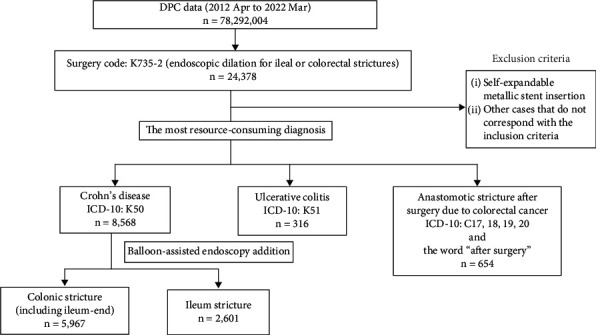
Study flowchart. Eligible admissions were extracted from the database as per this flowchart. DPC, Diagnosis Procedure Combination; ICD-10, International Classification of Diseases Tenth Revision.

We collected administrative claims data for all patients admitted to and subsequently discharged from more than 1000 DPC-participating hospitals from April 2012 to March 2022. We identified admissions involving EBD using Surgery Code K735-2. Notably, Surgery Code K735-2 encompasses endoscopic dilation for ileal or colorectal strictures and does not exclusively signify EBD. However, most cases of endoscopic dilation in Japan are estimated to be EBD. We classified eligible admissions into four categories: ileal stricture due to CD, colonic stricture due to CD, stricture due to UC, and anastomotic stricture due to surgery for intestinal cancers. CD and UC cases were identified using ICD-10 Codes K50 and K51, respectively, for the most resource-consuming diagnosis. Admissions following surgery for intestinal cancers were identified using ICD-10 Code C17, C18, C19, or C20, along with the word “after surgery” for the most resource-consuming diagnosis. Regarding CD-related admissions, we distinguished them by categorizing those involving the additional charge for balloon-assisted endoscopy as ileal strictures, whereas the remaining cases were classified as colonic strictures (including ileal-end strictures). Cases where self-expandable metallic stents were employed were excluded from the analysis (Figure 1).

Furthermore, we collected the following data from the DPC database: patient age, sex, BMI, smoking habits, and CCI [12]; hospital volume (the average number of EBD procedures per 1 year at each institution); medical costs; duration of hospital stay; and interventions after EBD (endoscopic hemostasis and urgent surgery: intestinal resection and stoma creation). We defined endoscopic hemostasis and urgent surgery on the same or next day after EBD as severe complications.

### 2.3. Statistical Analysis

Patient admissions were categorized according to age (≤ 49, 50–59, 60–69, 70–79, and ≥ 80 years) and BMI (underweight: < 18.5 kg/m^2^, normal weight: 18.5–24.9 kg/m^2^, and overweight: > 25.0 kg/m^2^), following the World Health Organization classification [13]. Smoking habits were classified into four categories using the Brinkman index [14]. The hospital volume was classified into four categories according to the average number of EBD procedures conducted annually at each institution: low, < 6.2; middle–low, < 16.4; middle–high, < 43.5; and high, ≥ 43.5.

We conducted a comparative analysis of the patients' backgrounds and clinical outcomes, including the number of EBD procedures in one admission, urgent surgery rate due to perforation, endoscopic hemostasis rate due to delayed bleeding, duration of hospital stay, and medical costs. We performed these comparisons in the following groups: (1) ileal and colonic stricture of CD, (2) strictures associated with CD and UC, and (3) strictures associated with CD and anastomotic strictures due to intestinal cancers. The chi-squared test or Wilcoxon's signed-rank test was used for the analyses. We also conducted propensity score matching using the following variables: sex, age, BMI, CCI, smoking history, and hospital volume.

Statistical significance was set at *p* < 0.05. All analyses were performed using JMP Pro16 software (SAS Institute, Tokyo, Japan). We calculated the C-statistics and standardized differences for each variable in propensity score matching.

### 2.4. Statement of Ethics

The study protocol was reviewed and approved by the Ethics Committee of the Tohoku University Graduate School of Medicine (2021-1-815). The need for informed consent was waived by the Ethics Committee of the Tohoku University Graduate School of Medicine.

## 3. Results

### 3.1. Background of the Study Population

We included 9538 eligible admissions in this study; 8568 were assigned to the CD group, whereas the remaining 316 and 654 were assigned to the UC and anastomotic stenosis groups, respectively. Although EBD for CD-related strictures exhibited no significant variance with regard to hospital volume, EBD for ileal strictures was performed more frequently in high-volume centers. Conversely, EBD for colonic strictures in CD and UC and anastomotic strictures after surgery due to intestinal cancers was conducted more frequently in low- and middle–low-volume centers (Table 1).

### 3.2. Clinical Practice of EBD for Strictures in CD and Comparisons Between Ileal and Colonic Strictures

Table 2 provides insights into the EBD outcomes for all CD admissions and shows the comparisons between ileal and colonic strictures in CD. Among the 8568 admissions within the CD group, 5967 were assigned to the colonic stricture group and the remaining 2601 to the ileal stricture group, based on the additional charge for balloon-assisted endoscopy. Most patients with CD underwent a single EBD procedure. The median duration of hospital stay was 4 days. The endoscopic hemostasis and urgent surgery rates after EBD for strictures associated with CD were considerably low (0.035% and 0.11%, respectively). Notably, EBD for ileal lesions was more frequently performed in high-volume centers.

Furthermore, the percentage of multiple EBD procedures within a single admission was higher for ileal strictures than for colonic strictures (3.6% vs. 2.7%, *p* = 0.039). The median duration of hospital stay for ileal strictures was shorter than that for colonic strictures. There were no significant differences in the severe complication rates between ileal and colonic strictures.

After propensity score matching, 2473 pairs were selected with a C-statistic of 0.67. All standardized differences for variables used in the propensity score matching were < 0.1, and the trends observed were consistent with those before propensity score matching.

### 3.3. Comparison of EBD Between CD and UC

The comparisons between CD and UC are summarized in Table 3. The rate of multiple EBD procedures for CD-related strictures was lower than that for UC-related strictures (3.0% vs. 5.1%, *p* = 0.045). Additionally, the median duration of hospital stay for CD admissions was shorter than that for UC admissions (4 vs. 5.5 days, *p* = 0.015). EBD for UC-related strictures was significantly more frequently performed in low-volume institutions compared with that for CD-related strictures (*p* < 0.0001). There were no significant differences in patient backgrounds or severe complication rates between the CD and UC groups.

After propensity score matching, 313 pairs were selected and the C-statistic was 0.73. All standardized differences of variables used in the propensity score matching were < 0.1. The rate of multiple EBD procedures for CD-related strictures was significantly lower than that for UC-related strictures (1.9% vs. 5.1%, *p* = 0.048). No significant differences were observed with regard to patient backgrounds, severe complication rates, and other clinical outcomes between the two groups.

### 3.4. Comparison of EBD Between CD and Anastomotic Strictures due to Intestinal Cancer Surgery

The results of comparisons between CD and anastomotic strictures after surgery for intestinal cancers are summarized in Table 4. The rate of multiple EBD procedures for CD-related strictures was lower than that for anastomotic strictures (3.0% vs. 5.5%, *p* = 0.0011). Additionally, the rates of urgent surgery and endoscopic hemostasis were lower for CD than those for anastomotic strictures due to cancer surgery (0.11% vs. 0.46% [*p* = 0.048] and 0.04 vs. 0.31% [*p* = 0.044], respectively). EBD for anastomotic strictures after cancer surgery was significantly more frequently performed in low-volume institutions compared to EBD for CD-related strictures (*p* < 0.0001). The duration of hospital stays of EBD for CD admissions was shorter than that for anastomotic strictures due to cancer surgery (4 vs. 5 days, *p* = 0.016). The median medical costs of EBD for CD admissions were higher than those for anastomotic strictures due to cancer surgery (JP¥346,340 vs. JP¥301,240, *p* = 0.032).

After propensity score matching, 378 pairs of admissions were analyzed and the C-statistic was 0.96. All standardized differences of variables used in the propensity score matching were < 0.1. Medical costs of CD-related EBD admissions were higher than medical costs of EBD for anastomotic strictures due to cancer surgery (JP¥385,360 vs. JP¥283,410, *p* < 0.0001). No significant differences were observed in the rate of multiple EBD procedures, patient backgrounds, duration of hospital stay, or severe complications between the two groups.

## 4. Discussion

In this study, we investigated the clinical practice of EBD for CD-related stricture. The median duration of hospital stay was 4 days, and the endoscopic hemostasis and urgent surgery rates after EBD for CD-related strictures were considerably low. Most patients with CD underwent only one EBD procedure during a single admission. Although there were no differences in patient backgrounds and the occurrence of severe complications between ileal and colonic strictures in CD, multiple EBD procedures were more frequently performed for ileal stricture than for colonic strictures. Furthermore, EBD for ileal stricture was prevalent in high-volume centers than in low-volume centers. Regarding severe complications after EBD, no significant differences were observed between CD-related strictures and UC or anastomotic stricture related to cancer surgery.

This study sheds light on the clinical practice of EBD for CD-related intestinal strictures. Most EBD procedures for CD-related strictures were conducted as a single intervention, irrespective of the specific location of the stricture. However, the frequency of EBD for small intestinal strictures in high-volume centers was notably higher compared with that for colonic strictures. This may indicate that EBD for small intestine strictures poses more technical challenges than that for colonic strictures. A retrospective study reported favorable EBD outcomes for colorectal strictures in patients with CD, with high technical success and low complication rates [15]. These findings align with those seen in EBD for small intestinal strictures [5, 7]. Despite the variation in hospital volume for EBD procedures, the clinical practice of EBD may be largely consistent between ileal and colonic strictures.

Our results indicate that EBD for CD-related intestinal stricture is extremely safe, with low complication rates, irrespective of whether the stricture is in the colon or ileum. However, the complication rates observed in this study are lower compared with perforation rates of 0%–10% reported in previous studies [4, 5, 7, 16–20]. This discrepancy may be attributed to the fact that we were unable to identify perforation and delayed bleeding directly owing to the nature of the DPC database. Therefore, we evaluated urgent surgery (indicative of perforation) and endoscopic hemostasis (indicative of delayed bleeding) as severe complications. Nevertheless, our findings and those of previous studies indicate that EBD is safe for the treatment of colonic and small intestinal strictures.

We also conducted a comparative analysis between EBD for CD- and UC-related strictures. EBD interventions for UC-related strictures often necessitated multiple procedures and were more frequently performed in low-volume centers as opposed to CD cases. Nevertheless, there were no significant differences in terms of safety and medical costs between the CD and UC cohorts. Despite the limited data on EBD for UC-related strictures, several studies have indicated that technical success and adverse event rates were comparable between the CD and UC groups [21, 22]. Notably, the number of patients with UC in these previous studies was considerably smaller than the corresponding CD cohorts [23, 24]. This may be attributed to the difference in the propensity for stricture formation between CD and UC. The incidence of intestinal stricture formation in patients with UC was significantly lower than that in patients with CD.

EBD for strictures resulting from cancer-related surgery demonstrated a higher complication rate and a need for multiple EBD procedures compared with EBD for CD-related strictures. However, the severe complication rate of EBD for strictures due to cancer-related surgery was low, similar to the rates associated with CD and UC. After propensity score matching, these differences were not significant. Several studies have reported favorable outcomes for EBD for strictures after colorectal surgery rather than IBD, with low complication rates [21, 22, 25, 26]. Furthermore, differences between the two groups disappeared.

This study had some limitations. First, as mentioned earlier, the DPC database lacks detailed clinical information, including laboratory data and endoscopic and computed tomography findings, which are essential for evaluating technical and clinical success and accurately assessing complications such as perforation and bleeding rates. Consequently, we relied on the evaluation of urgent surgery rates after EBD and endoscopic hemostasis as severe complications. Similarly, the DPC database did not contain information regarding details of EBD procedures (e.g., the number of strictures in one session of EBD, the reason of EBD, dilation diameter, and concomitant steroid injection) and characteristics of strictures (cause of strictures other than UC, CD, and cancer-related surgery). Although we performed a propensity score–matched analysis, biases cannot be completely denied. Therefore, our results should be interpreted with caution. Second, the DPC database does not track patients who are transferred to other hospitals. Therefore, we calculated the number of admissions rather than individual patients. Lastly, the DPC database primarily contains in-hospital data and lacks outpatient information (including treatment for UC and CD, as well as chemotherapy following surgery for colorectal cancers). Hence, we could not analyze the long-term outcomes after EBD.

In conclusion, our findings indicate that EBD for both ileal and colonic strictures in patients with CD can be performed safely, even though EBD for ileal strictures may be more challenging than that for colonic strictures. The clinical practice and safety of EBD for CD-related strictures were comparable to those for strictures resulting from other etiologies. Our findings highlight the safety and effectiveness of EBD in managing CD-related intestinal strictures.

## Figures and Tables

**Table 1:** Comparisons of clinical characteristics of the study population among underlying etiologies.

**Underlying etiology of stenosis**	**Crohn's disease**			**Ulcerative colitis**	**Anastomotic stenosis due to intestinal cancer**
	**All cases of Crohn's disease** **n** = 8568	**Colonic stricture (including terminal ileum)** **n** = 5967	**Ileum stricture** **n** = 2601	**n** = 316	**n** = 654
Sex (male/female)	6447/2121	4478/1489	1969/632	195/121	502/152
Mean age (SD) (years)	42.6 (12.1)	42.1 (12.0)	43.2 (12.5)	47.5 (16.7)	67.6 (10.2)
Age categories					
≥ 80 years	36	28	8	3	66
70–79 years	136	88	48	37	249
60–69 years	609	368	241	43	221
50–59 years	1511	1001	510	53	74
≤ 49 years	6276	4482	1794	180	44
Mean body mass index (SD) (kg/m^2^)	21.1 (9.9)	21.0 (11.7)	21.3 (3.5)	21.4 (4.4)	22.2 (3.6)
BMI categories					
Overweight (> 25.0 kg/m^2^)	1007	690	317	55	140
Normal range (18.5–24.9 kg/m^2^)	5442	3693	1749	181	426
Underweight (< 18.5 kg/m^2^)	2038	1534	504	77	83
Charlson comorbidity index score					
0	7111	4927	2184	226	407
1	1189	829	360	57	111
≥ 2	268	211	57	33	136
Brinkman index					
BI < 400	6905	4786	2119	254	321
400 ≤ BI < 600	422	303	119	10	44
600 ≤ BI < 1200	330	206	124	13	173
1200 ≤ BI	44	38	6	4	65
Hospital volume					
Low	2078	1751	327	100	490
Middle–low	2206	1668	538	117	136
Middle–high	2063	1348	715	73	20
High	2221	1200	1021	26	8

Abbreviations: BI, Brinkman index; BMI, body mass index; SD, standard difference.

**Table 2:** Comparisons of clinical characteristics of the study population and clinical outcomes between ileal and colonic stenoses.

	**All cases of Crohn's disease** **n** = 8568	**Before propensity score matching** **Total ** **n** = 8568			**After propensity score matching** **Total ** **n** = 4946			
		**Crohn's disease (colonic stricture including ileum end)** **n** = 5967	**Crohn's disease (ileal stricture)** **n** = 2601	**p** ** value**	**Crohn's disease (colonic stricture including ileum end)** **n** = 2473	**Crohn's disease (ileal stricture)** **n** = 2473	**p** ** value**	**Standardized difference**
Sex (male/female)	6447/2121	4478/1489	1969/632	0.53	1915/558	1887/586	0.36	0.027
Mean age (SD) (years)	42.6 (12.1)	42.1 (12.0)	43.2 (12.5)	< 0.0001	42.3 (11.8)	43.0 (12.1)	0.052	
Age categories				< 0.0001			0.73	
≥ 80 years	36	28	8		4	8		0.032
70–79 years	136	88	48		26	32		0.023
60–69 years	609	368	241		178	181		0.0047
50–59 years	1511	1001	510		477	477		0
≤ 49 years	6276	4482	1794		1788	1775		0.012
Mean body mass index (SD) (kg/m^2^)	21.1 (9.9)	21.0 (11.7)	21.3 (3.5)	< 0.0001	21.0 (3.4)	21.2 (3.5)	0.053	
BMI categories				< 0.0001			0.72	
Overweight (> 25.0 kg/m^2^)	1007	690	317		295	304		0.012
Normal range (18.5–24.9 kg/m^2^)	5442	3693	1749		1646	1645		0.00086
Underweight (< 18.5 kg/m^2^)	2038	1534	504		516	504		0.012
Charlson comorbidity index score				0.0043			0.18	
0	7111	4927	2184		2115	2086		0.033
1	1189	829	360		318	330		0.014
2	268	211	57		40	57		0.050
Brinkman index				0.0005			0.52	
BI < 400	6905	4786	2119		2044	2039		0.0053
400 ≤ BI < 600	422	303	119		96	113		0.034
600 ≤ BI < 1200	330	206	124		80	84		0.0090
1200 ≤ BI	44	38	6		3	6		0.028
Hospital volume				< 0.0001			0.97	
Low	2078	1751	327		332	327		0.0060
Middle–low	2206	1668	538		526	538		0.012
Middle–high	2063	1348	715		721	710		0.0098
High	2221	1200	1021		894	898		0.0034
Sessions of EBD during hospital stay				0.039			0.0013	
1	8311 (97.0%)	5803 (97.3%)	2508 (96.4%)		2420 (97.9%)	2381 (96.3%)		
≥ 2	257 (3.0%)	164 (2.7%)	93 (3.6%)		53 (2.1%)	92 (3.7%)		
Urgent surgery due to perforation after EBD, *n* (%)	9 (0.11%)	9 (0.15%)	0 (0%)	0.065	1 (0.04%)	0 (0%)	1.00	
Endoscopic hemostasis after EBD, *n* (%)	3 (0.035%)	2 (0.03%)	1 (0.04%)	1.00	0 (0%)	1 (0.04%)	1.00	
Median length of hospital stay (interquartile range) (days)	4 (3–9)	5 (3–10)	4 (3–6)	< 0.0001	4 (3–9)	4 (3–6)	< 0.0001	
Median medical costs of hospital stay (interquartile range) (JP¥)	346,340 (264,710–614,742.5)	344,140 (249,240–641,625)	348,410 (294,750–561,800)	0.35	331,770 (251,140–598,770)	348,300 (294,095–558,625)	0.018	
								C-statistic: 0.67

Abbreviations: BI, Brinkman index; BMI, body mass index; EBD, endoscopic balloon dilation; SD, standard difference.

**Table 3:** Comparisons of clinical characteristics of the study population and clinical outcomes between Crohn's disease and ulcerative colitis.

	**Before propensity score matching** **Total ** **n** = 8884			**After propensity score matching** **Total ** **n** = 626			
	**Crohn's disease** **n** = 8568	**Ulcerative colitis** **n** = 316	**p** ** value**	**Crohn's disease** **n** = 313	**Ulcerative colitis** **n** = 313	**p** ** value**	**Standardized difference**
Sex (male/female)	6447/2121	195/121	< 0.0001	185/128	193/120	0.57	0.028
Mean age (SD) (years)	42.6 (12.1)	47.5 (16.7)	< 0.0001	47.8 (15.4)	47.3 (16.6)	0.81	
Age categories			< 0.0001			0.93	
≥ 80 years	36	3		2	3		0.013
70–79 years	136	37		32	34		0.0070
60–69 years	609	43		50	43		0.021
50–59 years	1511	53		53	53		0
≤ 49 years	6276	180		176	180		0.0063
Mean body mass index (SD) (kg/m^2^)	21.1 (9.9)	21.4 (4.4)	0.097	21.2 (3.9)	21.4 (4.4)	0.55	
BMI categories			0.018			0.89	
Overweight (> 25.0 kg/m^2^)	1007	55		49	54		0.014
Normal range (18.5–24.9 kg/m^2^)	5442	181		187	179		0.012
Underweight (< 18.5 kg/m^2^)	2038	77		75	77		0.0047
Charlson comorbidity index score			< 0.0001			0.78	
0	7111	226		228	226		0.0028
1	1189	57		57	54		0.0082
2	268	33		28	33		0.018
Brinkman index			0.24			0.59	
BI < 400	6905	254		259	251		0.011
400 ≤ BI < 600	422	10		12	10		0.012
600 ≤ BI < 1200	330	13		7	13		0.038
1200 ≤ BI	44	4		2	4		0.023
Hospital volume			< 0.0001			0.99	
Low	2078	100		97	99		0.00041
Middle–low	2206	117		119	115		0.0076
Middle–high	2063	73		72	73		0.0024
High	2221	26		25	26		0.0040
Sessions of EBD during hospital stay			0.045			0.048	
1	8311 (97.0%)	300 (94.9%)		307 (98.1%)	297 (94.9%)		
≥ 2	257 (3.0%)	16 (5.1%)		6 (1.9%)	16 (5.1%)		
Urgent surgery due to perforation after EBD, *n* (%)	9 (0.11%)	1 (0.32%)	0.30	0 (0%)	1 (0.32%)	1.00	
Endoscopic hemostasis after EBD, *n* (%)	3 (0.035%)	0 (0%)	1.00	0 (0%)	0 (0%)	—	
Median length of hospital stay (interquartile range) (days)	4 (3–9)	5.5 (3–19)	0.015	5 (3–11)	6 (3–19)	0.15	
Median medical costs of hospital stay (interquartile range) (JP¥)	346,340 (264,710–614,742.5)	365,950 (244,797.5–1,015,718)	0.64	374,380 (269,755–679,175)	364,650 (245,100–1,013,620)	0.68	
							C-statistic: 0.73

Abbreviations: BI, Brinkman index; BMI, body mass index; EBD, endoscopic balloon dilation; SD, standard difference.

**Table 4:** Comparisons of clinical characteristics of the study population and clinical outcomes between Crohn's disease and anastomotic stenosis due to intestinal cancer.

	**Before propensity score matching** **Total ** **n** = 9222			**After propensity score matching** **Total ** **n** = 756			
	**Crohn's disease** **n** = 8568	**Anastomotic stricture due to intestinal cancer** **n** = 654	**p** ** value**	**Crohn's disease** **n** = 378	**Anastomotic stricture due to intestinal cancer** **n** = 378	**p** ** value**	**Standardized difference**
Sex (male/female)	6447/2121	502/152	0.40	276/102	275/103	1.00	0.0060
Mean age (SD) (years)	42.6 (12.1)	67.6 (10.2)	< 0.0001	61.5 (12.4)	63.2 (10.4)	0.034	
Age categories			< 0.0001			0.97	
≥ 80 years	36	66		20	16		0.019
70–79 years	136	249		75	74		0.0024
60–69 years	609	221		168	170		0.0032
50–59 years	1511	74		71	74		0.0072
≤ 49 years	6276	44		44	44		0
Mean body mass index (SD) (kg/m^2^)	21.1 (9.9)	22.2 (3.6)	< 0.0001	21.6 (3.9)	21.9 (3.5)	0.22	
BMI categories			< 0.0001			0.96	
Overweight (> 25.0 kg/m^2^)	1007	140		66	68		0.0050
Normal range (18.5–24.9 kg/m^2^)	5442	426		249	244		0.0068
Underweight (< 18.5 kg/m^2^)	2038	83		61	63		0.0052
Charlson comorbidity index score			< 0.0001			0.71	
0	7111	407		268	270		0.0026
1	1189	111		50	55		0.014
≥ 2	268	136		60	53		0.019
Brinkman index			< 0.0001			0.90	
BI < 400	6905	321		215	217		0.0029
400 ≤ BI < 600	422	44		28	30		0.0075
600 ≤ BI < 1200	330	173		78	79		0.0023
1200 ≤ BI	44	65		22	16		0.028
Hospital volume			< 0.0001			0.56	
Low	2078	490		234	247		0.018
Middle–low	2206	136		107	103		0.0080
Middle–high	2063	20		29	20		0.037
High	2221	8		8	8		0
Sessions of EBD during hospital stay			0.0011			1.00	
1	8311 (97.0%)	618 (94.5%)		363 (96.0%)	362 (95.8%)		
≥ 2	257 (3.0%)	36 (5.5%)		15 (4.0%)	16 (4.2%)		
Urgent surgery due to perforation after EBD, *n* (%)	9 (0.11%)	3 (0.46%)	0.048	0 (0%)	2 (0.53%)	0.50	
Endoscopic hemostasis after EBD, *n* (%)	3 (0.04%)	2 (0.31%)	0.044	2 (0.53%)	1 (0.26%)	1.00	
Median length of hospital stay (interquartile range) (days)	4 (3–9)	5 (3–16)	0.016	5 (3–12)	5 (3–15)	0.35	
Median medical costs of hospital stay (interquartile range) (JP¥)	346,340 (264,710–614,742.5)	301,240 (219,505–853,770)	0.032	385,360 (264,630–704,390)	283,410 (217,900–853,790)	< 0.0001	
							C-statistic: 0.96

Abbreviations: BI, Brinkman index; BMI, body mass index; EBD, endoscopic balloon dilation; SD, standard difference.

## Data Availability

The DPC data used to support the findings of this study were supplied by the Japanese Ministry of Health, Labour and Welfare under approval and so cannot be made freely available. Requests for access to these data should be made to the corresponding author.
